# Insanity and Allied Neuroses

**Published:** 1908-04

**Authors:** 


**Insanity and Allied Neuroses.** A Practical and Clinical Manual. By **George H. Savage,** M.D., F.R.C.P., late Physician and Superintendent of Bethlehem Royal Hospital. With the assistance of **Edwin Goodall,** M.D. (Lond.) Medical Superintendent of the Cardiff City Hospital for Mental Diseases. 12 mo. pp. 638. Illustrated. Fourth Edition. Chicago: W. T. Keener & Co. (Price, $2.75.)

A fourth edition of Savage on Insanity needs no formal introduction to the medical profession, as its author is one of the best known of English alienists, and a man for whom all have a profound respect for his opinions. The present edition has been revised throughout and shows the tendency of the times, by paying considerably more attention than in former editions to the pathological substratum of mental diseases. In this direction, Doctor Savage has enlisted the services of Dr. Mott, which is assurance enough that the work has been well and scientifically done.

In the opening chapters the author discusses insanity from the legal and medical standpoints—pointing out the difference between insanity, eccentricity, genius, precocity, and then takes up the relationship of crime to mental unsoundness. The author believes that such a relationship exists and that other nervous diseases are very apt to appear in criminals. He mentions epilepsy especially, also sense perversions.

Regaring the classification of the insanities, the author does not follow in the lead of the German alienists, Kralpelin for instance, and discard melancholia, mania, and circular insanity, but in the old familiar way discusses the different forms of melancholia, the various groups of mania, and folie circulaire, just as if manic-depressive insanity had never been heard of.

The dementias are carefully considered, but dementia precox is not given the same hearty indorsement that the German and American alienists accord it. The English, and Savage as their spokesman, have always insisted on the retention of moral insanity in the classification of the insanities, and the chapter on this form is interesting and debatable. Sexual perversions, sexual inversion Waster's, those addicted to false accusations, cruelty and vindictiveness, depraved tastes and the like are or may be degenerates—moral degenerates if you will, but hardly irresponsible in a medical or legal sense. They are amenable to the law and not strictly unaccountable for their acts. The different groups of insanity are treated in a careful painstaking manner, and make enjoyable reading of a most difficult and obtuse subject. The illustrations, especially the colored ones, are well executed and add much to the worth of the book. It is bound in flexible covers and may be conveniently carried in the pocket. W. C. K

